# An investigation of the predictors of photoprotection and UVR dose to the face in patients with XP: a protocol using observational mixed methods

**DOI:** 10.1136/bmjopen-2017-018364

**Published:** 2017-08-21

**Authors:** Jessica Walburn, Robert Sarkany, Sam Norton, Lesley Foster, Myfanwy Morgan, Kirby Sainsbury, Vera Araújo-Soares, Rebecca Anderson, Isabel Garrood, Jakob Heydenreich, Falko F Sniehotta, Rute Vieira, Hans Christian Wulf, John Weinman

**Affiliations:** 1 Institute of Pharmaceutical Science, King’s College London, London, UK; 2 St John’s Institute of Dermatology, Guy’s and St. Thomas’ NHS Foundation Trust, London, UK; 3 Health Psychology Section, Institute of Psychiatry, Psychology & Neuroscience, King’s College London, London, UK; 4 Institute of Health & Society, Faculty of Medical Science, Newcastle University, Newcastle, UK; 5 Newcomen Centre, Guy’s and St. Thomas’ NHS Foundation Trust, London, UK; 6 Department of Dermatology, Bispebjerg Hospital, Copenhagen, Denmark

**Keywords:** adherence, dosimeter, mixed methods, photoprotection, rare conditions, Xeroderma pigmentosum

## Abstract

**Introduction:**

Xeroderma pigmentosum (XP) is a rare genetic condition caused by defective nucleotide excision repair and characterised by skin cancer, ocular and neurological involvement. Stringent ultraviolet protection is the only way to prevent skin cancer. Despite the risks, some patients’ photoprotection is poor, with a potentially devastating impact on their prognosis. The aim of this research is to identify disease-specific and psychosocial predictors of photoprotection behaviour and ultraviolet radiation (UVR) dose to the face.

**Methods and analysis:**

Mixed methods research based on 45 UK patients will involve qualitative interviews to identify individuals’ experience of XP and the influences on their photoprotection behaviours and a cross-sectional quantitative survey to assess biopsychosocial correlates of these behaviours at baseline. This will be followed by objective measurement of UVR exposure for 21 days by wrist-worn dosimeter and daily recording of photoprotection behaviours and psychological variables for up to 50 days in the summer months. This novel methodology will enable UVR dose reaching the face to be calculated and analysed as a clinically relevant endpoint. A range of qualitative and quantitative analytical approaches will be used, reflecting the mixed methods (eg, cross-sectional qualitative interviews, n-of-1 studies). Framework analysis will be used to analyse the qualitative interviews; mixed-effects longitudinal models will be used to examine the association of clinical and psychosocial factors with the average daily UVR dose; dynamic logistic regression models will be used to investigate participant-specific psychosocial factors associated with photoprotection behaviours.

**Ethics and dissemination:**

This research has been approved by Camden and King’s Cross Research Ethics Committee 15/LO/1395. The findings will be published in peer-reviewed journals and presented at national and international scientific conferences.

**Strengths and limitations of this study**

- This is the first investigation of photoprotection in patients with xeroderma pigmentosum (XP).
- We use a novel mixed methods approach to investigate predictors of photoprotection behaviours in XP.
- We have created an innovative method to calculate dose of ultraviolet radiation reaching the face.
- Recruitment of participants may be a challenge, although a feature of research in rare diseases.

## Introduction

Xeroderma pigmentosum (XP) is a rare autosomal recessive inherited condition caused by defective nucleotide excision repair. The incidence is 2.3 per million live births in Western Europe.^1^ Patients may develop skin cancers from childhood onwards, ocular damage and neurological deterioration^2^ and many patients suffer abnormal severe and easy sunburn reactions.^3^ The phenotype is variable and strongly dependent on the complementation group and on the mutations.^2^ Lifespan varies between countries and in USA the median age at death is 32 years, the main cause of death being skin cancer.^4^ The clinical management of XP relies on rigorous photoprotection which is the only means of preventing skin cancer and eye disease. To date, no research has been conducted to ascertain whether *rigorous* photoprotection is achieved by patients. Adherence to photoprotection is poor in non-XP survivors of malignant melanoma^5^ and anecdotal evidence from clinicians caring for patients with XP suggests that patients with XP may vary widely in the degree to which they photoprotect.

A recent review of photoprotection in immunosuppressed patients highlights the gap between knowledge of photoprotection recommendations and behaviour.^6^ That the generic provision of knowledge is not enough to change behaviour has been identified in recent reviews of adherence interventions across chronic conditions.^7^ Recent research has therefore focused on identifying modifiable psychosocial determinants of behaviour. A key focus has been the perceptions a person holds about their illness^8^ and treatment.^9^ These form a personal belief model which influences disease-related behaviour, including adherence to treatment.^10^ It is also possible, due to the heterogeneity of XP, that there may be disease-related differences between and within patients that could affect photoprotection behaviour either directly or via the beliefs held about the condition. We anticipate that poor ultraviolet radiation (UVR) protection in patients with XP may be critically dependent on such psychological, social and disease-related factors and that these may be amenable to intervention.

This research uses a mixture of qualitative and quantitative methods to assess levels of photoprotection in this population—exploring individuals’ experiences of XP and influences on photoprotection behaviours, differences between and within individuals over time and individual differences in clinical and psychosocial factors. Though the typical ‘large sample’ epidemiological studies are not possible in such a rare condition, the combination of methods contributes a comprehensive understanding and gives a unique viewpoint allowing for a whole person perspective for the variation in photoprotection. This will enable the development of individually tailored interventions to improve photoprotection and thus improve outcomes.

To investigate whether poor adherence is associated with worse medical outcomes, the research needs to incorporate a clinical measure. As the causal link between UVR exposure and cancer incidence is not contentious,^11^ we will focus on UVR dose to the face as the most clinically relevant end point, combining personal UVR exposure measured by UVR dosimetry with a self-reported record of photoprotection behaviours throughout the day. The research will have a meaningful clinical outcome and provides an opportunity to test a novel photodermatological measurement approach.

### Objectives

1. To explore individuals’ experiences of XP and influences on photoprotection behaviours.
2. To objectively measure UVR exposure and by adjusting for photoprotection behaviours, calculate the UVR dose to the face.
3. To identify the psychological, social, disease-specific and sociodemographic factors associated with dose of UVR to the face.
4. To identify psychological and social predictors of within-individual variation in photoprotection behaviours over time.

## Methods and analysis

### Design

The research uses mixed methods: cross-sectional qualitative interviews and self-report questionnaire, longitudinal diary study of photoprotection behaviours and potential predictors (n-of-1) for 50 days, estimation of UVR dose to the face for 21 days.

### Participants

A purposive sample of patients diagnosed with XP will be recruited from the caseload of the XP specialist service at Guy’s & St Thomas’ NHS Foundation Trust, which is composed of the majority of known cases with XP in the UK (n=93). The aim is to recruit 45 participants—25 adults and adolescents who are responsible for their own photoprotection behaviour and 20 participants consisting of children and younger adolescents, plus cognitively impaired individuals where a carer is responsible for photoprotection. Individuals will be eligible to participate if they have a confirmed diagnosis of XP and are aged between 1 and 85 years. Those with inadequate English to take part in an interview and not currently living in the UK will be excluded. Given the number of with people known to have XP, the sample size is based on what is deemed feasible rather than on statistical power calculations.

### Procedure

A research nurse will contact and recruit eligible patients by sending them an invitation letter and participant information sheet. The main carer will be contacted for patients younger than 16 years of age and adults who lack the capacity to consent due to XP-related cognitive impairment. Age appropriate information sheets will be provided. The patients will be called 2 weeks later to see if they are interested in taking part. Those with a routine appointment at the XP clinic will be approached at the clinic. Patients wishing to participate will complete written informed consent.

**Figure 1:**
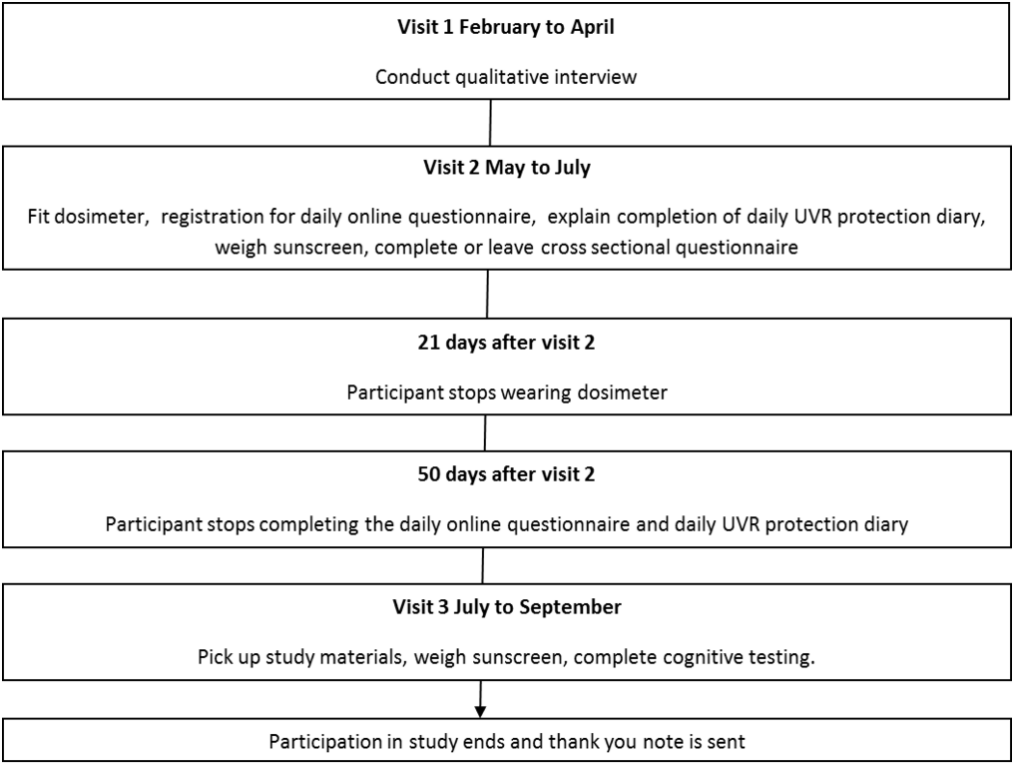
Diagram of data collection protocol (2016, 2017). UVR, ultraviolet radiation.

As shown in figure 1, adult participants will be visited by research staff a maximum of three times. The following describes the detailed procedure of the adult protocol in order of visit. Visits will occur at a convenient time for the participant although visit 2 will be between May and mid-July (in both 2016 and 2017) to ensure that dosimeters are recording when UVR levels are typically at their highest in the UK. Visit 3 will occur after all assessments have been completed.

#### Visit 1:

Semistructured interviews will be conducted to gain a comprehensive understanding of the meanings of XP and the influences on individuals’ photoprotection behaviours including perceptions of risk, experiences of stigma and the role and processes of informal social support. Interviews will take place in a private room in the patient’s house and participants will be interviewed on their own by a researcher, unless they are under 16 years when they will be interviewed with a parent or carer. Carers of adults with cognitive impairment will be interviewed if patients are unable to take part due to the extent of their impairment. A topic guide will cover the participant’s personal story of XP, nature of the burden, photoprotection behaviours, impact of XP on everyday life, perception of UVR risk and social environment. The topic guide will provide a flexible framework to structure the discussion but the direction of the interview will be led by the participant.

#### Visit 2

The nurse will complete the registration process for the daily online (n-of-1) questionnaire and will explain how to use the UVR protection diary. Both of these daily assessments need to be completed every evening for 50 days. They will be given the dosimeter and instructed to wear it when they go outside for a minimum of 21 days. This ‘dosimetry period’ was shorter than the 50-day ‘daily photoprotection period’ (assessment of predictors and behaviours) due to concerns from the Patient and Public Involvement (PPI) panel about participant burden. Participants will have the opportunity to wear the dosimeter for up to 50 days if they wish. To obtain a measurement of routine use of sunscreen the participant will be supplied with their preferred brand which will be weighed before use. Participants will be instructed to only use this sunscreen during the dosimetry period. Participants will be given a cross-sectional questionnaire to complete during the visit or in the period between visits 2 and 3.

#### Visit 3

Materials will be collected and used sunscreen bottles will be weighed to calculate the amount used. The anatomical location (ie, face, neck, hands) of the sunscreen used will be recorded. Research staff will conduct cognitive tests. XP-related data will be collected from clinical files held at the XP service. To avoid unnecessary participant burden, participants younger than 16 years and those with cognitive impairment will complete fewer assessments across fewer visits, the daily online (n-of-1) questionnaire will not be completed and fewer cognitive tests will be conducted. Carers will complete the UVR protection diary and the cross-sectional questionnaire about the participant, if they are either too young or lack the capacity to do it themselves.

#### Materials

(a) The UVR electronic dosimeter (SunSaver 3, Bispebjerg Hospital, Copenhagen, Denmark) is worn on the wrist and provides real-time measurements of the UVR level in the patient’s environment.^12^ It has been shown to be a reliable measure of personal UVR exposure behaviour in healthy individuals^13^ and melanoma survivors.^5^ It measures UVR exposure (SEDs), movement (number of movements) and temperature (°C) every 5 s and records the mean every 5 min.

(b) The daily online (n-of-1) questionnaire is accessed through a SMART phone or an internet-linked computer. Text messages containing a link to the online questionnaire are delivered daily to the participant’s phone/device using SurveySignal, at their preferred time; the survey is administered using the Qualtrics software (Qualtrics, Provo, Utah, USA) platform. It uses touch screen technology with all questions using a slider scale.

(c) The daily UVR protection diary will be used to record time spent outside, specific photoprotection behaviours and the type of outdoor activity. The protection diary was based on an adapted version of the UK Office of National Statistics Time Use Survey,^14^ with the aim of improving the reliability and validity of the diary formats used in previous studies which require a dichotomous response to a question about each day (eg, *Did you use sunscreen today ?* Y/N), by recording the duration of each behaviour. It is a paper diary where each page represents 1 day. It has a grid format with the day split into 15 min segments starting from 06:00 to 22:00 hours to ensure all daylight hours are included. Participants will record their time spent outside (>10 min) rounded to the nearest 15 min (ie, between 10 and 15 min are recorded as 15 min) and their photoprotection behaviours for the face (visor, hat, hoodie worn-up, glasses, scarf or face buff, sunscreen, lip-block) during that time. Participants will draw a line to represent the amount of time they wore each clothing item and tick when they applied sunscreen or lip-sunblock. This will allow multiple behaviours to be recorded for the same time period. To understand what people were doing when they were outside, participants will record activities against a prespecified list (eg, socialising with friends). A copy of the UVR protection diary is included in the online supplementary appendix 1.

10.1136/bmjopen-2017-018364.supp1 Supplementary file 1

### Measures

#### Predictor variables

##### Demographic and clinical data

A range of XP-related variables likely to affect photoprotection behaviours and therefore UVR dose will be collected from medical records. This includes XP complementation group (genotype, DNA repair activity), the presence or absence of XP-related cognitive impairment, severity of eye and neurological disease, severity of sunburn,^3^ cancer number and type of skin and eye cancers, age at diagnosis (clinical and when confirmed by laboratory testing), years since diagnosis and age when photoprotection started. Demographic data (age, gender, ethnicity, presence or absence of family member with XP) will be collected.

##### Cognitive ability

To investigate the impact of general cognitive ability and higher level thinking processes on photoprotection behaviours, cognitive testing will be undertaken using a standardised test of IQ (Wechsler Abbreviated Scale of Intelligence (WASI-II))^15^ and two tests sensitive to executive functioning (Delis-Kaplan Executive Function System).^16^ The two subtest version of the WASI-II (FSIQ-2, Vocabulary and Matrix Reasoning) will be used to give an estimate of general intellectual functioning in participants aged 6 years or older and with sufficient cognitive capacity to engage in testing. The verbal fluency (phonemic and semantic) and the Tower of London tasks will test response generativity, planning and monitoring, in participants aged 16 years or older.

##### Psychological and social factors

A variety of psychological and social variables will be assessed by a cross-sectional self-report questionnaire. As no prior research has investigated predictors of photoprotection in XP, the variables have been selected on the basis of relevant psychological theories and research examining photoprotection in healthy populations.^17^

1. Perceptions of XP: Adapted Brief Illness Perception Questionnaire,^18^ measuring perceptions of XP (consequences, timeline, personal and treatment control, identity, coherence and emotional response) on a 0–10 scale.
2. Treatment beliefs: Adapted version of Beliefs about Medicine Questionnaire^19^ to assess beliefs about the necessity of photoprotection and concerns about photoprotecting. Respondents state the extent to which they agree with statements on a five-item scale.
3. Intention (motivation), self-efficacy (confidence) and habit: These factors are assessed for each photoprotection behaviour by recording the strength of agreement with statements (eg, *I intend to wear a visor; I am confident I could wear a visor; Every time I got ready to go outside, wearing a visor was something I did automatically without thinking*) on a 7-point scale. Intention and self-efficacy items are adapted from a manual for the design of questionnaires based on the Theory of Planned Behaviour.^20^ Habit items are adapted from the Self-Report Habit Index.^21^
4. Social support: The level of support and satisfaction with it is recorded using two items adapted from Social Support Questionnaire^22^ on a 5-point scale *(eg, How satisfied are you with the support or help that you have to help you with your UV protection? Very dissatisfied to very satisfied).*
5. Emotional well-being, quality of life and time perspective (ie, if decisions are based on present or future consequences) will be measured using standard scales: Short-form Warwick-Edinburgh Mental Well-Being Scale (SWEMWBS),^23^ EQ-5D-5L,^24^ Adapted Zimbardo Time Perception Inventory.^25 26^

The questionnaire includes disease-related factors, financial costs of XP to the participant and a measure of adherence to photoprotection behaviours (these data will be analysed as part of an international survey of predictors of photoprotection in XP and will be used to validate responses on the UVR protection diary) (see online supplementary appendix 2).

10.1136/bmjopen-2017-018364.supp2 Supplementary file 2

A range of psychological and social variables are measured in the daily online (n-of-1) questionnaire. Questions will be selected as described in (3), informed by the content of initial qualitative interviews and on the likelihood of variation over time (eg, stress). The following environmental, physical and psychological (cognitive and emotional) constructs, as related to UVR protection, will be measured: perception of weather, symptoms, rumination, social support, negative consequences (eg, missing out), effort, automaticity, goal conflict, self-consciousness, mental exhaustion, stress, energy, mood and quality of life, all reported retrospectively at the end of the day. Prospective questions related to photoprotection on the next day will assess motivation, confidence, goal priority and planning. The stem ‘*Thinking about protecting your face from UVR when you went outside today/go outside tomorrow…’* preceded each question; for example, ‘*How much stress has it caused you?’* All questions were answered on a 0–100 sliding scale (see online supplementary appendix 3).

10.1136/bmjopen-2017-018364.supp3 Supplementary file 3

### Outcome variables

#### Mean daily UVR dose to the face (standard erythema dose (SED))

The face is the site of the overwhelming majority of skin cancers in XP, so the most clinically relevant measure is the dose of UVR to facial skin. This will be captured by combining data from the UVR dosimeter device worn on the wrist, with participants’ UVR protective behaviours recorded in the UVR protection diary and the weight of sunscreen used during the study period. Combining dosimeter and diary is an approach that has been used to validate self-report diary data of sun exposure behaviour,^27^ to relate personal UVR exposure to different activities (eg, work, leisure),^28^ and to calculate site-specific dose.^29^ However, the outcome in those studies was the dose at the wrist, whereas this study will combine that data with the photoprotection behaviours recorded on the UV protection diary, to calculate the proportion of the environmental UV dose which reaches the face.

The dosimeter measures the SED every 5 s and gives the average of these measurements every 5 min. These are combined to provide total UVR exposure for the 15-min intervals relating to the periods specified in the activity diary. During each 15-min interval, the dose of UVR to the face (in SEDs) equals the UVR exposure recorded by the dosimeter weighted by the protection associated with photoprotection behaviours recorded for that interval on the daily UVR protection diary.

Weights will be generated based on the degree of photoprotection afforded by each photoprotection practice, informed by a review of published literature and photodermatology expert judgement. To account for protective behaviours selectively protecting different areas, the weights are produced by separating the face into five different regions. This comprised the forehead (upper third), nose and cheeks (middle third), chin and jaw (lower third), eyes and lips. Each of the three facial segments contributes 30% towards the photoprotection weighting for the whole face, with lips and eyes contributing 5% each. Where no protection is used, the weight given is 1 and the UVR dose to the face equals the total UVR exposure for the interval. Virtually no UVR in the range 290–400 nm was measured to penetrate the UVR protective visor (unpublished data). Therefore, where a visor is indicated to have been worn, since it protects all five regions of the face, the weight assigned is 0 and the UVR dose to the face is 0 SED. Other combinations of behaviours provide selective coverage to different regions—for example, glasses were assumed to protect only the eyes and a scarf or face buff the lower third of the face and lips. Protection provided by a hat is modelled based on the position of the sun in the sky relative to the person’s home address. Given the latitude of the UK, a hat with typical brim effectively provides protection only to the forehead.

The level of protection provided by sunscreen to all thirds of the face and lip block to the lips is modelled separately, with a reducing function over time. Given previous research indicates that sunscreen is typically applied at 20%–50% of the 2 mg/cm^2^ thickness required to achieve the stated sun protection factor (SPF),^11^ we will assume patients with XP apply sunscreen at approximately 40% thickness (ie, 0.8 mg/cm^2^). Since all individuals will be provided with broad-spectrum sunscreen SPF 50+, and there is a square-root association between thickness and SPF, we will assume that 20.8% of UVR exposure reached the face at the time sunscreen is initially applied. The level of protection is conservatively assumed to reduce following a linear function with no protection provided 8 hours after initial application. The assumptions concerning thickness will be examined by comparison with the average amount of sunscreen used by each participant, as measured by weighing the tubes of sunscreen provided at visit 1.

#### Adherence to photoprotection advice using the daily online (n-of-1) questionnaire and UVR protection diary

Adherence to photoprotection advice will be assessed as a standalone outcome using both self-report (single item: *How much have you protected your face from UVR?*) on the online daily questionnaire and the daily photoprotection behaviours reported in the UVR protection diary. The clinical team will be asked to estimate the adequacy of the protection provided by different combinations of behaviours (eg, hat and glasses). Participants will also be asked to report their satisfaction with their protection on the online questionnaire.

### Analysis

As shown in table 1, the different data collection methods will be used and combined as required to achieve the research objectives. Analysis for calculation of UVR dose to the face is described in Outcomes I.

**Table 1:** Combination of data collection methods for study objectives

	Objectives			
	To explore individuals’ experience of XP and influences on photoprotection behaviours.	To objectively measure UVR exposure and by adjusting for photoprotection behaviours, calculate the UVR dose to the face.	To identify the psychological and social, disease-specific and sociodemographic factors associated with dose of UVR to the face.	To identify psychological and social predictors of within-individual variation in photoprotection behaviours over time.
Qualitative interviews	X			
Cross-sectional questionnaire			X	
Daily online (n-of-1) questionnaire				X
Daily UVR protection diary		X		X
Sunscreen weight		X		
Dosimeter		X		
Cognitive tests			X	
Sociodemographic and clinical data			X	

UVR, ultraviolet radiation; XP, xeroderma pigmentosum.

#### To explore individuals’ experience of XP and influences on photoprotection behaviours

The qualitative analysis will provide an in-depth exploration of individuals ‘experience of photoprotection behaviours in a population with a lack of previous research. All interviews will be audio-recorded, transcribed and entered into NVivo10. A thematic ‘framework’ analysis will be undertaken which will involve a detailed examination of patterns within and across cases. This requires an iterative dynamic process of consistently testing and refining themes and explanations and involves ongoing discussions between researchers. The validity of emerging explanations and categories will be examined through triangulation based on discussion groups held with clinical staff of the XP service.

#### To identify the psychological, social and disease-specific factors associated with dose of UVR to the face in people with XP

When examining the UVR dose to the face, the total UVR exposure and estimated UVR dose to the face are summed so that the unit of analysis will be the total level per day for each individual. Mixed-effects longitudinal models will examine the association of clinical and psychosocial factors with the average daily UVR dose. Given that daily UVR exposure and dose to the face are unlikely to be normally distributed, an appropriate generalised model will be used (eg, lognormal). Analyses will control for total daily environmental UVR (provided by the Public Health England Solar Network monitoring station with the shortest geodetic distance to the participant’s home address (mean 43.3 miles)) and will be stratified by whether the photoprotection diary was completed by the participant or their carer. Since the level of adherence to photoprotection, in terms of the proportion of UVR exposure protected from reaching the face, is the ratio of estimated UVR dose to the face to the total UVR exposure, the parameter estimates from models for each outcome will be combined to provide an indication of the effect of the predictor on photoprotection adherence. This allows for consideration of whether the effect of the predictor is on the total UVR exposure, which is driven by time spent outside, or UVR dose to the face, which is driven by time spent outside *and* photoprotection behaviours while outside.

#### To identify psychological and social predictors of within-individual variation in photoprotection behaviours over time

The analysis of within-participant variability in photoprotection behaviours over time (n-of-1 methodology^30^) achieves statistical power by the number of repeated observations (ie, completion of daily online questionnaire and photoprotection diary) throughout time, which in this type of analysis represents the sample size.^31^ The analysis will identify factors that may result in fluctuation in an individual’s photoprotection adherence and provides a fine-grained understanding of the within-person variation in photoprotection behaviours and the psychological and social predictors of these changes. Objective protection will be dichotomised for each person to reflect their ‘best’ protection (defined as the highest level of protection achieved and used on at least 10 occasions over the study period) versus the rest (ie, behaviours that are less than best). The correspondence between objective protection used in the first 15 min of each outdoor occasion and each predictor will be analysed using dynamic logistic regression,^32^ controlling for study day, the order of multiple outdoor occasions within the same day and past behaviour (photoprotection used on each of the previous two outdoor occasions). Dynamic regression uses the past to explain the future by including in the model variables that represent the predictor, as reported that outdoor occasion (lag 0) as well as on the previous occasion(s) (eg, previous occasion—lag 1; two occasions prior—lag 2). This allows for the identification of potential delayed effects of these predictors on photoprotection. For participants with limited or no variability in photoprotection behaviour, visual inspection of the predictors will instead be used.

## Discussion

To the best of our knowledge, this is the first investigation of photoprotection in patients with XP. The mixed methods approach will provide detailed knowledge of the nature and predictors of a hitherto poorly understood complex set of behaviours, linked to a novel biologically relevant outcome in XP. It will advance existing approaches used to measure adherence to photoprotection by combining objective assessment of UVR from a dosimeter with self-report data of photoprotection from a diary to estimate the dose of UVR that reaches the skin on the face. Potential predictors from a range of data sources are assessed (eg, cognitive tests, self-report psychological variables measured cross-sectionally and daily) and combined.

The multimethods approach will provide a unique and rich understanding of the processes underpinning and influencing photoprotection behaviours. These findings will be used to inform the development of a behaviour change intervention designed to improve photoprotection. The additional insight provided by mixed methods research is recommended by published frameworks developed to guide systematic intervention development (eg, Intervention Mapping^33^). A consensus conference, attended by researchers, PPI representatives and the XP clinical team, will be undertaken to decide which of the identified predictors are modifiable and will be targeted in a series of individually tailored interventions to improve photoprotection in patients with XP.

It is acknowledged that participant recruitment to a study involving multiple data collection procedures might be challenging. This is particularly pertinent considering the rareness of XP whereby half of the UK XP population is required to participate. However, the research team have worked closely with the PPI Panel and XP clinical team adapting the protocol to ensure that the study presents a tolerable and practical ‘patient burden’. It is anticipated that the findings of this research will be generalisable to other conditions requiring photoprotection or a high degree of adherence to complex behaviours.

## Ethics and dissemination

This research has been approved by Camden and King’s Cross Research Ethics Committee 15/LO/1395. The findings will be published in peer reviewed journals and presented at national and international scientific conferences.

## Supplementary Material

Reviewer comments
