# Supplementary material for: An investigation of the predictors of photoprotection and UVR dose to the face in patients with XP: a protocol using observational mixed methods
Source: BMJ Open. 2017 Aug 21;7(8):e018364. doi: 10.1136/bmjopen-2017-018364 (PMC5724163; doi:10.1136/bmjopen-2017-018364)
Supplement: Supplementary file 1 [file bmjopen-2017-018364supp001.pdf]

Appendix 1. The UVR protection diary.

UV diary

B = By myself, outdoors
G = Gardening
R = Repairs
P = Playing sports, exercising or going for a walk
SH = Shopping or errands
Socialising:
SF = Socialising with family
SFR = Socialising with friends

Travelling (e.g., walk/train/bus/car):
TS = Travelling to/from social or leisure activity
TW = Travelling to/from work, school or college
TO = Travelling to/from other
W = Work, school or college related activity
O = Other

1 Date (day of week) ☐

2 Did you go outside today? (Please tick) YES ☐ NO ☐ If no, skip to question 4

3 Let us know when you were outside by marking a line on the diary

	6am	7am	8am	9am	10am	11am	12pm	1pm	2pm	3pm	4pm	5pm	6pm	7pm	8pm	9pm
I was outside																

4 Let us know if you wore any of these things today by marking a line on the diary

	6am	7am	8am	9am	10am	11am	12pm	1pm	2pm	3pm	4pm	5pm	6pm	7pm	8pm	9pm
Hat																
Glasses																
Scarf or facebuff																
Hoodie worn up																
Face visor																

5 If you put sunscreen on your face or used lip sun block tell us each time you did this by ticking the diary

	6am	7am	8am	9am	10am	11am	12pm	1pm	2pm	3pm	4pm	5pm	6pm	7pm	8pm	9pm
Sun screen																
Lip sunblock																

6 If you did not wear or use any of these things today tick in the box ☐

7 Did you go outside between 10pm last night and 6am today? (Please tick) YES ☐ NO ☐

IF YES what time did you go out? ☐

What time did you return? ☐

Comments

7 DAYS | 14 DAYS | 21 DAYS | 28 DAYS | 35 DAYS | 42 DAYS | 50 DAYS
