# Supplementary material for: An investigation of the predictors of photoprotection and UVR dose to the face in patients with XP: a protocol using observational mixed methods
Source: BMJ Open. 2017 Aug 21;7(8):e018364. doi: 10.1136/bmjopen-2017-018364 (PMC5724163; doi:10.1136/bmjopen-2017-018364)
Supplement: Supplementary file 2 [file bmjopen-2017-018364supp002.pdf]

Appendix 2.

XP N-of-1 questions - FINAL

Note: questions answered using a 0-100 sliding scale (not at all – completely; very little – a great deal) unless otherwise indicated

Question group	Question
Thinking about the weather today…	How sunny has it been?
	How much did you need to protect yourself from UV?
Quality of life (alone)	How would you rate your quality of life today? (very poor – very good)
Thinking generally about your need for UV protection today…	How much have negative thoughts about XP got stuck in your head?
	How much have you felt supported by other people?
	How much has it made you feel that you were missing out?
	How much have you felt any physical symptoms/effects of UV? (e.g., skin soreness, redness, itching, headaches, uncomfortable eyes etc.)
Thinking generally about your feelings today – not necessarily related to XP or UV protection…	How mentally exhausted have you felt?
	How active have you felt?
	How would you describe your mood today? (very negative – very positive)
Screener (alone)	Did you go outside during daylight today?
Thinking about protecting your face from UV when you went outside today…	How much have you protected your face from UV?
	How much effort have you put into it?
	How much did you need to think about doing it?
	How much have other things got in the way of it?
	How much stress has it caused you?
	How self-conscious have you felt?
	How happy have you been with your level of UV protection?
Thinking about protecting your face from UV when you go outside tomorrow...	How motivated are you?
	How confident are you?
	How important is it compared to other things you want/need to do?
	How much have you planned to make sure you do it?
