# Supplementary material for: An investigation of the predictors of photoprotection and UVR dose to the face in patients with XP: a protocol using observational mixed methods
Source: BMJ Open. 2017 Aug 21;7(8):e018364. doi: 10.1136/bmjopen-2017-018364 (PMC5724163; doi:10.1136/bmjopen-2017-018364)
Supplement: Supplementary file 3 [file bmjopen-2017-018364supp003.pdf]

Appendix 3.

Participant study code_______________ Country of residence: UK

Understanding Ultraviolet Protection (UV) in Xeroderma Pigmentosum (XP) Questionnaire

Xeroderma Pigmentosum National Service
2nd Floor, South Wing
St Thomas' Hospital
Westminster Bridge Road
London SE1 7EH

Guy's and St Thomas' NHS
NHS Foundation Trust

We are trying to understand more about how having to protect against daylight affects the lives of people who have XP. From the answers we hope to be able to find ways to help patients and their families.
Please answer as many questions as you can. Where you are not sure of an answer, it is fine if you put your best guess.

Remember all the information you give will be treated with the strictest confidence and will be analysed anonymously. The people who look after you in the XP clinic will not see the answers you give.

If you are 12-15 years old and take responsibility for your own UV protection, please look through the questionnaire FIRST to see whether you feel able to complete it on your own. If you do not think you can, or if you are unsure, it is best for your parent or carer to complete one for you instead. They will complete a different version of questionnaire. Please contact our Research Nurse,(*************) if you do not have the version for parents or carers.

Thank you for taking the time to answer this questionnaire. Because XP is rare, every questionnaire that is completed and sent back to us makes a big difference for this research.

Filling out the questionnaire takes about 25 minutes. There are questions on both sides of each page.

The questionnaire is split into different topics:

- **XP and your thoughts about it**
- **What you do to protect yourself from Ultraviolet (UV) in daylight**

  Some questions ask about what you usually do and others ask about the last few days or weeks. Some questions look the same as others but are actually different, so please can you try to answer as accurately as you can.
- **Your views about UV protection**
- **General things about you and your quality of life**

Before we ask about your XP, we'd like to find out a few details about you.

1. How old are you now? _______________Years

2. Are you: (please tick) Male ☐ or Female ☐

3. I am completing the questionnaire because: (please tick one option)

I have been diagnosed with XP and I am 16 years or over	
I have been diagnosed with XP and I am 12 - 15 years old and have chosen to complete the questionnaire myself	

4. Do you have any of these qualifications? (Please tick all that apply). If you have no formal qualifications please tick the box below

NVQ or equivalent	
GCE / GCSE / O-level or equivalent	
A-level or equivalent	
Pre-degree foundation course/ diploma / HND or equivalent	
Undergraduate degree	
Masters	

Appendix 3.

Participant study code_______________ Country of residence: UK

Doctorate/ PhD	
Professional qualification	
Other<br>Please write in________________________	
No Formal qualifications	

ABOUT YOUR XP

Please complete or circle the answer

5. How old were you when you were diagnosed as having XP?	…….….…. Years
6. Have you ever had a skin cancer?	Yes / No
7. Have you had to see an Eye Specialist doctor because of any problems with the eyes?	Yes / No
8. Has the XP caused problems with your hearing, walking, or speaking?	Yes / No
9. Do you sunburn more easily than other people in your family who do not have XP?	Yes / No
10. Have you ever had sunburn so badly you needed to see a doctor about it?	Yes / No
11. Have you ever had sunburn in the winter in the country where you live?	Yes / No
12. Have you ever had sunburn which took more than 3 days to go away?	Yes / No
13. Do you take special measures to avoid some light bulbs?	Yes / No

14. How would you describe your skin?	
	Fair or light coloured
	Asian
	Light brown
	Afrocaribbean
	Dark brown
15. What colour are your eyes?	
	Blue
	Green
	Brown
	Other
16. Do you know what complementation group of XP you have?	Yes / No
17. If **Yes** please circle the group	
	A
	B
	C
	D
	E
	F
	G
	V

We are interested in your own personal views of how you see all aspects of your XP.

Appendix 3.

Participant study code_______________ Country of residence: UK

Please circle the number that best corresponds to your views:

18. How much does your XP affect your life?<br><br>0 1 2 3 4 5 6 7 8 9 10<br>*no affect at all* … *severely affects my life*
19. How long do you think your XP will continue?<br><br>0 1 2 3 4 5 6 7 8 9 10<br>*a very short time* … *forever*
20. How much control do you feel you have over the way XP affects your health?<br><br>0 1 2 3 4 5 6 7 8 9 10<br>*Absolutely no control* … *extreme amount of control*
21. How much do you think daylight/UV protection can help your skin or eye health?<br><br>0 1 2 3 4 5 6 7 8 9 10<br>*not at all* … *extremely helpful*
22. How much do you think XP treatment in the clinic (e.g. surgery, creams) can help your skin or eye health?<br><br>0 1 2 3 4 5 6 7 8 9 10<br>*not at all* … *extremely helpful*
23. How much do you experience symptoms related to your XP?<br><br>0 1 2 3 4 5 6 7 8 9 10<br>*no symptoms at all* … *many severe symptoms*
24. How concerned are you about your XP?<br><br>0 1 2 3 4 5 6 7 8 9 10<br>*not at all concerned* … *extremely concerned*

Please circle the number that best corresponds to your views:

25. How well do you feel you understand your XP?

0	1	2	3	4	5	6	7	8	9	10
don't understand at all										*understand very clearly*

26. How much does your XP affect you emotionally? (does it make you angry, scared, upset or depressed?)

0	1	2	3	4	5	6	7	8	9	10
not at all affected emotionally										*extremely affected emotionally*

YOU HAVE COMPLETED SECTION 1
YOU'RE ALMOST A THIRD OF THE WAY THROUGH

Appendix 3.

Participant study code________________ Country of residence: UK

ABOUT YOUR PROTECTION FROM ULTRAVIOLET (UV) IN DAYLIGHT

27. What is the date today? (day/month/year)	…………./…………../…………….

28. Which is your nearest town?<br><br>**<u>If you do not wish to give this information please move to the next question.</u>**	

These questions focus on the <u>LAST 7 DAYS</u>

29. Over the last 7 days, what is your best guess for **<u>how many days</u>** it has been sunny for at least half the day?	……………………….Days (out of 7)

30. Thinking about a **<u>typical day</u>** in the last 7 days, what is your best guess on average for the **<u>number of hours</u>** you have spent outside in daylight (with or without UV protection)?	…………………hours per day

31. We know it can be difficult to protect against UV all the time. Over the last 7 days when you went outside how often did you protect yourself against UV?<br>(Please circle the answer)	Never<br>rarely<br>sometimes<br>often<br>always

32. Do you have UV protective window film on home windows?<br>(Please circle the answer)	I have protective film on:<br>ALL windows<br>SOME windows<br>NO windows<br>I don't know

33. Over the last 7 days, when at home to what extent have you	Not at all

stayed in rooms with UV protection<br>(Please circle the answer)	rarely<br>sometimes<br>often<br>always

34. Is there UV protective window film on windows at school or work?<br>(Please circle the answer)	There is film on:<br>ALL windows<br>SOME windows<br>NO windows<br>I don't know<br>I don't work or go to school outside the home

35. Over the last 7 days, when at work or school to what extent have you stayed in rooms with UV protection<br>(Please circle the answer)	Not at all<br>rarely<br>sometimes<br>often<br>always

36. Do you have UV protective window film on your car's windows?<br>(Please circle the answer)	There is film on:<br>ALL windows<br>SOME windows<br>NO windows<br>I don't know<br>I don't have a car

Appendix 3.

Participant study code_______________ Country of residence: UK

We know it can be difficult to protect against UV in daylight all the time.

These items ask how much you agree or disagree with the statements about the different things you could do to protect yourself from UV.

They are very similar statements about each way you could have protected yourself over the PAST 7 DAYS. Please read each question carefully and circle the number that best corresponds to your views. If you did not protect from UV in that way please circle the box "I did not…"

OVER THE LAST 7 DAYS…..

37. ***"Avoiding going outside*** *during the day was something I did automatically without thinking"*<br>1 2 3 4 5 6 7<br>Strongly Disagree … Strongly Agree	I did not avoid going outside
38. *"Every time I got ready to go outside,* ***wearing a face visor*** *was something I did automatically without thinking"*<br>1 2 3 4 5 6 7<br>Strongly Disagree … Strongly Agree	I did not wear a face visor
39. *"Every time I got ready to go outside,* ***wearing a hat*** *was something I did automatically without thinking"*<br>1 2 3 4 5 6 7<br>Strongly Disagree … Strongly Agree	I did not wear a hat
40. *"Every time I got ready to go outside* ***wearing glasses*** *was something I did automatically without thinking"*<br>1 2 3 4 5 6 7<br>Strongly Disagree … Strongly Agree	I did not wear glasses
41. *"Every time I got ready to go outside,* ***wearing sunscreen*** *was something I did automatically without thinking"*<br>1 2 3 4 5 6 7<br>Strongly Disagree … Strongly Agree	I did not wear sunscreen

42. *"Every time I got ready to go outside,* ***wearing lip sunblock*** *was something I did automatically without thinking"*<br><br>1 2 3 4 5 6 7<br>Strongly Disagree Strongly Agree	I did not wear lip sunblock
43. *"Every time I got ready to go outside,* ***wearing a scarf or "face buff"*** *was something I did automatically without thinking"*<br><br>1 2 3 4 5 6 7<br>Strongly Disagree Strongly Agree	I did not wear a scarf or "face buff"
44. *"Every time I got ready to go outside,* ***wearing a hoodie (worn up)*** *was something I did automatically without thinking"*<br><br>1 2 3 4 5 6 7<br>Strongly Disagree Strongly Agree	I did not wear a hoodie (worn-up)
45. *"Every time I got ready to go outside,* ***wearing long sleeves**** *was something I did automatically without thinking"*<br><br>1 2 3 4 5 6 7<br>Strongly Disagree Strongly Agree	I did not wear long sleeves
46. *"Every time I got ready to go outside,* ***wearing gloves*** *was something I did automatically without thinking"*<br><br>1 2 3 4 5 6 7<br>Strongly Disagree Strongly Agree	I did not wear gloves
47. *"Every time I got ready to go outside,* ***wearing long trousers or thick tights*** *was something I did automatically without thinking"*<br><br>1 2 3 4 5 6 7<br>Strongly Disagree Strongly Agree	I did not wear long trousers or thick tights

***sleeves would include coats or jackets**

OVER THE LAST 7 DAYS WHEN OUTSIDE…..

48. Thinking about all the things you did to protect yourself over the past 7 days (e.g., wearing sunscreen, wearing a hat), how well do you think they protected you from UV? (Please circle).<br><br>*Completely* *very well* *a fair amount* *a little* *Not at all*	I did not do anything to protect myself from UV in the last 7 days

Appendix 3.

Participant study code_______________ Country of residence: UK

We know that people adapt their UV protection to fit in with everyday life.

These questions are about what you USUALLY do throughout the year to protect yourself from UV in daylight when it is CLOUDY AND then what you do on days when it is SUNNY.

Please circle how often you do the different things on the days when it is CLOUDY and then on the days when it is SUNNY, throughout the year.

49. To what extent do you try to avoid going outside during the day?	**Cloudy days**	**Sunny days**
	Never<br>rarely<br>sometimes<br>often<br>always	Never<br>rarely<br>sometimes<br>often<br>always

WHEN YOU GO OUTSIDE… (Please circle the answer)

	**Cloudy days**	**Sunny days**
**Thinking about your FACE….**		
50. How often do you wear a face visor?	Never<br>rarely<br>sometimes<br>often<br>always	Never<br>rarely<br>sometimes<br>often<br>always
51. How often do you wear a hat?	Never<br>rarely<br>sometimes<br>often<br>always	Never<br>rarely<br>sometimes<br>often<br>always
52. How often do you wear glasses?	Never<br>rarely<br>sometimes<br>often<br>always	Never<br>rarely<br>sometimes<br>often<br>always

**When you go outside ……<br>(Please circle the answer)**	**Cloudy days**	**Sunny days**
53. How often do you use sunscreen on your face?	Never<br>rarely<br>sometimes<br>often<br>always	Never<br>rarely<br>sometimes<br>often<br>always
54. If you use sunscreen on your face, what sun protection factor does it have?<br>(or circle I do not….)	SPF number ………	SPF number ………
	I do not wear sunscreen on my face	
55. How often do you reapply the sunscreen on your face during the day?<br>(or circle I do not….)	……………..x/day	……………..x/day
	I do not wear sunscreen on my face	
56. How often do you put on lip sunblock?	Never<br>rarely<br>sometimes<br>often<br>always	Never<br>rarely<br>sometimes<br>often<br>always
57. How often do you wear a scarf or “face buff”?	Never<br>rarely<br>sometimes<br>often<br>always	Never<br>rarely<br>sometimes<br>often<br>always

Appendix 3.
Participant study code________________ Country of residence: UK

When you go outside …… (Please circle the answer)	Cloudy days	Sunny days
58. How often do you wear a hoodie (worn -up)?	Never<br>rarely<br>sometimes<br>often<br>always	Never<br>rarely<br>sometimes<br>often<br>always
**Thinking about your ARMS OR HANDS…**		
59. How often do you wear long sleeves?	Never<br>rarely<br>sometimes<br>often<br>always	Never<br>rarely<br>sometimes<br>often<br>always
60. How often do you use sunscreen on your arms or hands?	Never<br>rarely<br>sometimes<br>often<br>always	Never<br>rarely<br>sometimes<br>often<br>always
61. How often do you wear gloves?	Never<br>rarely<br>sometimes<br>often<br>always	Never<br>rarely<br>sometimes<br>often<br>always
**Thinking about your LEGS…**		
62. How often do you wear long trousers or thick tights?	Never<br>rarely<br>sometimes<br>often<br>always	Never<br>rarely<br>sometimes<br>often<br>always

When you go outside ……<br>(Please circle the answer)	Cloudy days	Sunny days
63. How often do you use sunscreen on your legs?	Never<br>rarely<br>sometimes<br>often<br>always	Never<br>Rarely<br>sometimes<br>often<br>always
**Thinking about using sunscreen on your BODY (NOT FACE), ARMS, HANDS OR LEGS….**		
64. If you use sunscreen on any of these areas (body, arms, hands or legs) what sun protection factor does it have? (or circle I do not….)	SPF number ………	SPF number ………
	I do not wear sunscreen on my body, arms, hands or legs	
65. How often do you reapply the sunscreen to any of these areas (body, arms, hands or legs) during the day? (or circle I do not….)	……………..x/day	……………..x/day
	I do not wear sunscreen on my body, arms, hands or legs	

YOU'RE OVER HALF OF THE WAY THROUGH NOT MUCH MORE TO GO..

Appendix 3.

Participant study code_______________ Country of residence: UK

We would like to ask your personal views about protecting against UV in daylight. This means whatever you do to protect yourself, including:

- **avoiding going outside during the day**
- **putting on sunscreen and lip sunblock**
- **wearing glasses**
- **wearing protective clothes (e.g., hat, scarf or face-buff, gloves, long sleeves, long trousers/thick tights)**
- **or wearing a face visor**

These are statements other people have made about their UV protection.

Please indicate the extent to which you agree or disagree with what they have said by circling the answer that best corresponds to your views.

There are no right or wrong answers. We are interested in <u>YOUR PERSONAL VIEWS.</u>

66. *"My health depends on protecting against UV"*<br>Strongly Agree Agree Uncertain Disagree Strongly Disagree
67. *"Having to protect against UV worries me"*<br>Strongly Agree Agree Uncertain Disagree Strongly Disagree
68. *"My life would be impossible without protecting against UV"*<br>Strongly Agree Agree Uncertain Disagree Strongly Disagree
69. *"If I did not protect against UV I would be very ill"*<br>Strongly Agree Agree Uncertain Disagree Strongly Disagree
70. *"I sometimes worry about other people's reactions to the things I have to do to protect against UV (e.g., wearing a visor, using sunscreen, wearing a hat)"*<br>Strongly Agree Agree Uncertain Disagree Strongly Disagree
71. *"I sometimes worry about long-term effects of having to protect against UV"*<br>Strongly Agree Agree Uncertain Disagree Strongly Disagree

72. *"I don't really understand why I need to protect against UV"*

Strongly Agree Agree Uncertain Disagree Strongly Disagree
73. *"My health in the future will depend on protecting against UV"*<br>Strongly Agree Agree Uncertain Disagree Strongly Disagree
74. *"Having to protect against UV disrupts my life"*<br>Strongly Agree Agree Uncertain Disagree Strongly Disagree
75. *"Protecting against UV stops my health getting worse"*<br>Strongly Agree Agree Uncertain Disagree Strongly Disagree
76. *"I sometimes worry about the impact on family and friends of having to protect against UV"*<br>Strongly Agree Agree Uncertain Disagree Strongly Disagree

We are now asking about the NEXT 7 DAYS.

These questions are about the different things you might do to protect yourself from UV. We know that people want to do some things more than others, and they find some things easier to do than others. We want to find out what YOU THINK.

These statements are very similar to each other about the ways you might protect yourself over the next 7 days. Please read each question carefully and think about each way in turn.

77. Over the next 7 days I intend to protect myself by **avoiding going outside** during the daytime<br>1 2 3 4 5 6 7<br>Strongly Disagree Strongly Agree

Appendix 3.

Participant study code________________ Country of residence: UK

When I am outside in the NEXT 7 DAYS….

(If you are not intending to go outside in the next 7 days please skip to question 88.)

78. I intend to protect myself by **wearing a face visor**

Strongly Disagree 1	2	3	4	5	6	7 Strongly Agree

79. I intend to protect myself by **wearing a hat**

Strongly Disagree 1	2	3	4	5	6	7 Strongly Agree

80. I intend to protect myself by **wearing glasses**

Strongly Disagree 1	2	3	4	5	6	7 Strongly Agree

81. I intend to protect myself by **putting on sunscreen**

Strongly Disagree 1	2	3	4	5	6	7 Strongly Agree

When I am outside in the NEXT 7 DAYS….

82. I intend to protect myself by **putting on lip sunblock**

Strongly Disagree 1	2	3	4	5	6	7 Strongly Agree

83. I intend to protect myself by **wearing a scarf or "face buff"**

Strongly Disagree 1	2	3	4	5	6	7 Strongly Agree

84. I intend to protect myself by **wearing a hoodie (worn-up)**

Strongly Disagree 1	2	3	4	5	6	7 Strongly Agree

85. I intend to protect myself by **wearing long sleeves**

Strongly Disagree 1	2	3	4	5	6	7 Strongly Agree

86. I intend to protect myself by **wearing gloves**

Strongly Disagree 1	2	3	4	5	6	7 Strongly Agree

87. I intend to protect myself by **wearing long trousers or thick tights**

Strongly Disagree 1	2	3	4	5	6	7 Strongly Agree

"We know these questions are repetitive…thank you for keeping going"

Over the NEXT 7 DAYS…..

88. I am confident I could **avoid going outside** during the daytime
1 2 3 4 5 6 7
Strongly Disagree ... Strongly Agree

When I am outside in the NEXT 7 DAYS….
(If you are not intending to go outside in the next 7 days please skip to question 99.)

89. I am confident I could **wear a face visor**
1 2 3 4 5 6 7
Strongly Disagree ... Strongly Agree
90. I am confident I could **wear a hat**
1 2 3 4 5 6 7
Strongly Disagree ... Strongly Agree
91. I am confident I could **wear glasses**
1 2 3 4 5 6 7
Strongly Disagree ... Strongly Agree

When I am outside in the NEXT 7 DAYS….

92. I am confident I could put on **sunscreen**
1 2 3 4 5 6 7
Strongly Disagree ... Strongly Agree
93. I am confident I could put on **lip sunblock**
1 2 3 4 5 6 7
Strongly Disagree ... Strongly Agree
94. I am confident I could **wear a scarf or "face buff"**
1 2 3 4 5 6 7
Strongly Disagree ... Strongly Agree
95. I am confident I could **wear a hoodie (worn-up)**
1 2 3 4 5 6 7
Strongly Disagree ... Strongly Agree

Appendix 3.

Participant study code________________ Country of residence: UK

96. I am confident I could **wear long sleeves**

1 2 3 4 5 6 7

Strongly Disagree Strongly Agree

97. I am confident I could **wear gloves**

1 2 3 4 5 6 7

Strongly Disagree Strongly Agree

98. I am confident I could easily **wear long trousers or thick tights**

1 2 3 4 5 6 7

Strongly Disagree Strongly Agree

Overall in the NEXT 7 DAYS………

99. I am confident I can do the things I want to do to protect myself from UV

1 2 3 4 5 6 7

Strongly Disagree Strongly Agree

About your XP and the people around you……

100. How much support or help do you have from the people around you with your UV protection?

No support *hardly any support* *Some support* *lots of support* *comprehensive support*

101. How satisfied are you with the support or help that you have to help you with your UV protection?

Very dissatisfied *fairly dissatisfied* *uncertain* *fairly satisfied* *very satisfied*

YOU'RE NOW IN THE FINAL SECTION
YOU'VE ALMOST FINISHED !

ABOUT YOU AND YOUR QUALITY OF LIFE

These questions are about you, your thoughts about the present and future, your emotions and your quality of life.

Please circle the number that best corresponds to your views:

102. *"Thinking about the future is pleasant to me"*						
	1	2	3	4	5	
Very true of me						Very untrue of me
103. *"When I want to achieve something, I set goals and consider specific means of reaching those goals"*						
	1	2	3	4	5	
Very true of me						Very untrue of me
104. *"Meeting tomorrow's deadlines and doing other necessary work comes before tonight's play"*						
	1	2	3	4	5	
Very true of me						Very untrue of me
105. *"It seems to me that my future plans are pretty well laid out"*						
	1	2	3	4	5	
Very true of me						Very untrue of me
106. *"I think that it seems useless to plan too far ahead because things hardly ever come out the way you planned anyway"*						
	1	2	3	4	5	
Very true of me						Very untrue of me
107. *"If I do not get done on time, I do not worry about it"*						
	1	2	3	4	5	
Very true of me						Very untrue of me
108. *"I try to live one day at a time"*						
	1	2	3	4	5	
Very true of me						Very untrue of me
109. *"I feel that it is more important to enjoy what you are doing than to get the work done on time"*						
	1	2	3	4	5	
Very true of me						Very untrue of me
110. *"I do not do things that are good for me if they do not feel good now"*						
	1	2	3	4	5	
Very true of me						Very untrue of me

Appendix 3.

Participant study code_______________ Country of residence: UK

Below are some statements about feelings and thoughts.

Please circle the answer that best describes your experience of each over the LAST 2 WEEKS

111.	*"I've been feeling optimistic about the future"*				
	None of the time	Rarely	Some of the time	Often	All of the time
112.	*"I've been feeling useful"*				
	None of the time	Rarely	Some of the time	Often	All of the time
113.	*"I've been feeling relaxed"*				
	None of the time	Rarely	Some of the time	Often	All of the time
114.	*"I've been dealing with problems well"*				
	None of the time	Rarely	Some of the time	Often	All of the time
115.	*"I've been thinking clearly"*				
	None of the time	Rarely	Some of the time	Often	All of the time
116.	*"I've been feeling close to other people"*				
	None of the time	Rarely	Some of the time	Often	All of the time
117.	*"I've been able to make up my own mind about things"*				
	None of the time	Rarely	Some of the time	Often	All of the time

These questions are about how your quality of life is affected by the XP. Under each heading, please tick the ONE box that best describes your health TODAY.

118. MOBILITY

I have no problems in walking about ❑

I have slight problems in walking about ❑

I have moderate problems in walking about ❑

I have severe problems in walking about ❑

I am unable to walk about ❑

119. SELF-CARE

I have no problems washing or dressing myself ❑

I have slight problems washing or dressing myself ❑

I have moderate problems washing or dressing myself ❑

I have severe problems washing or dressing myself ❑

I am unable to wash or dress myself ❑

120. USUAL ACTIVITIES (e.g. work, study, housework, family or leisure activities)

I have no problems doing my usual activities ❑

I have slight problems doing my usual activities ❑

I have moderate problems doing my usual activities ❑

I have severe problems doing my usual activities ❑

I am unable to do my usual activities ❑

121. PAIN / DISCOMFORT

I have no pain or discomfort ❑

I have slight pain or discomfort ❑

I have moderate pain or discomfort ❑

I have severe pain or discomfort ❑

I have extreme pain or discomfort ❑

<u>Appendix 3</u>.

Participant study code_______________ Country of residence: UK

122. ANXIETY / DEPRESSION

I am not anxious or depressed ❑

I am slightly anxious or depressed ❑

I am moderately anxious or depressed ❑

I am severely anxious or depressed ❑

I am extremely anxious or depressed ❑

- We would like to know how good or bad your health is TODAY.
- This scale is numbered from 0 to 100.
- 100 means the <u>best</u> health you can imagine.
  0 means the <u>worst</u> health you can imagine.
- Mark an X on the scale to indicate how your health is TODAY.
- Now, please write the number you marked on the scale in the box below.

123. **YOUR HEALTH TODAY** = [ ]

The best health you can imagine

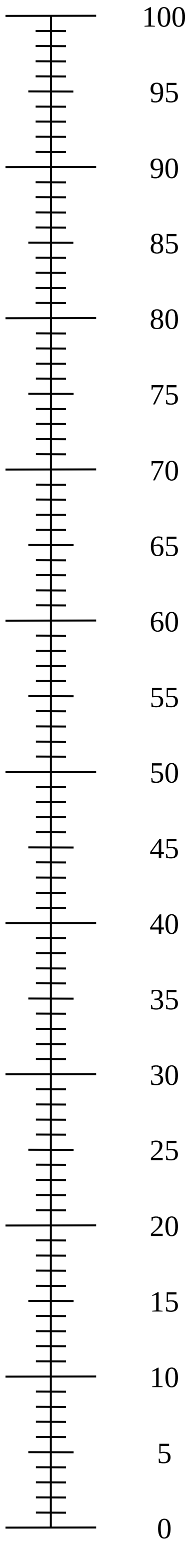

The worst health you can imagine

Appendix 3.

Participant study code_______________ Country of residence: UK

ONE MORE QUESTION ABOUT YOU…

This is an extra question, some people may not want to fill it out. If you don't want to do this question, just leave it blank.

124. Which of the following **best** describes your **ethnicity?** Please tick **one** of the following options.

**White**<br>British	☐	**Black or Black British**<br>Caribbean	☐
Any other White background<br>please write in___________________	☐	African	☐
**Mixed**<br>White and Black Caribbean<br>White and Black African<br>White and Asian<br>Any other Mixed background<br>please write in___________________	☐<br>☐<br>☐<br>☐	Any other Black background,<br>please write in__________________<br><br>**Chinese or other ethnic group**<br>Chinese<br>Any other<br>Please write in___________________	☐<br><br><br><br>☐<br>☐
**Asian or Asian British**			
Indian	☐		
Pakistani	☐		
Bangladeshi	☐		
Any other Asian background<br>please write in___________________	☐		

If you have any additional thoughts or comments please can you write them here:

THANK YOU VERY MUCH FOR COMPLETING OUR QUESTIONNAIRE.
