# Supplementary material for: An investigation of the predictors of photoprotection and UVR dose to the face in patients with XP: a protocol using observational mixed methods
Source: BMJ Open. 2017 Aug 21;7(8):e018364. doi: 10.1136/bmjopen-2017-018364 (PMC5724163; doi:10.1136/bmjopen-2017-018364)
Supplement: Reviewer comments [file bmjopen-2017-018364.reviewer_comments.pdf]

PEER REVIEW HISTORY

BMJ Open publishes all reviews undertaken for accepted manuscripts. Reviewers are asked to complete a checklist review form (**http://bmjopen.bmj.com/site/about/resources/checklist.pdf**) and are provided with free text boxes to elaborate on their assessment. These free text comments are reproduced below.

ARTICLE DETAILS

**TITLE (PROVISIONAL)**	An investigation of the predictors of photoprotection and UVR dose to the face in patients with XP: A protocol using observational mixed methods
**AUTHORS**	Walburn, Jessica; Sarkany, Robert; Norton, Sam; Foster, Lesley; Morgan, Myfanwy; Sainsbury, Kirby; Araújo-Soares, Vera; Anderson, Rebecca; Garrood, Isabel; Heydenreich, Jakob; Sniehotta, Falko; Vieira, Rute; Wulf, Hans Christian; Weinman, John

This article was not externally reviewed at BMJ Open. Protocols that have been independently assessed prior to submission to BMJ Open are usually fast-tracked to publication on the grounds that further substantial changes will not be possible. This independent assessment will usually be external, independent review for both a substantial grant award from a non-commercial or government funder and ethics approval.
